# Concluding commentary: Medical Education in Difficult Circumstances

**DOI:** 10.15694/mep.2017.000062

**Published:** 2017-03-24

**Authors:** Michelle McLean, Trevor Gibbs, Judy McKimm

**Affiliations:** 1 Faculty of Health Sciences & Medicine; 2 AMEE; 3 College of Medicine

**Keywords:** Difficult Circumstances, Medical and Health Professions' Education

## Abstract

This article was migrated. The article was marked as recommended.

We live in difficult times. As three medical educators with many years of experience in medical education in different contexts and in a range of countries, we recognise that many educators and students face ‘difficult circumstances’. Over the 18 months or so, including a theme at the 2016 AMEE Conference in Barcelona, we have collaborated with colleagues and students to identify some of our ‘wicked’ problems as well as possible solutions in medical and health professions’ education. This Commentary summarises the contexts, difficulties and actual or potential solutions (e.g. low-resource settings, adverse weather events) described by collleagues who submitted articles to the January-March themed edition of *MedEdPublish.* In most of the submissions, we identified resourceful interventions and frameworks for tackling ‘difficult circumstances’. Over the next few months, we will collate the information we have collected with the view to offering a range of frameworks and collaborative opportunities to support colleagues requiring assistance.

## Introduction

The idea of a themed edition in *MedEdPublish* (January-March 2017) emerged from the positive response to the various contributions (oral and poster presentations, plenary, symposium, workshop) in the *“Medical Education in Difficult Circumstances”* theme at the 2016 AMEE Conference in Barcelona. At the Conference, we were able to develop a consensual definition of what constitutes a ‘difficult circumstance’ and identify a range of such circumstances, which were summarised in the Editorial introducing the *MedEdPublish* theme ( Gibbs et al., 2017).

Although there were several follow-up emails to presenters in this theme at AMEE 2016, we received only seven contributions that met the criteria. Four submissions dealt with the challenges of low-resource settings ( Artemiou & Adams, 2017; Mack et al., 2017; Tibyampansha et al., 2017; Morikawa, 2017) while, in a similar vein, Cartwright and colleagues’ (2017) submission offered twelve tips for adapting the curriculum in times of economic austerity. One submission addressed the bedside as a ‘difficult’ learning environment ( Biswas & Tzadok, 2017) while two papers dealt with providing medical education in adverse weather events ( Niedl et al., 2017; Barzansky & Hash, 2017).

Unlike the 2016 AMEE meeting where the focus was primarily to identify problems, the seven submissions in the *MedEdPublish* theme were generally ‘good news’ stories in that the authors offered frameworks or solutions (often resourceful) for addressing their ‘difficulties’. It is interesting to note that one of the papers dealt with communication skills in veterinary education. We acknowledge that the issues identified by the authors are no different from those faced in many of health professions’ contexts.

## Synopsis of the submissions

The four papers describing opportunities and adaptations in low-resource settings reflect different contexts and problems. For Artemiou and colleagues’ (2017), ‘low-resource’ relates to the remoteness of their offshore veterinary college (Ross University) on the island of St. Kitts, which, with Nevis, is considered a ‘developing’ (low-income) nation. Although their ‘difficulty’ was structurally related to improving communication skills, their *MedEdPublish* submission covers issues such as culture and multi-culturalism, learning in remote settings, a curriculum split between two very different countries (the Caribbean islands and mainland USA) and a lack of available resources. Useful tips on dealing with these issues are provided as well as highlighting the benefits of the location and resources for both students and staff. No doubt there will be further papers appearing from these authors as the School develops. In the online discussion that followed one of the editor’s (MM) review of the paper, it was interesting to learn that some graduates return to practice as veterinarians on St. Kitts. This is a positive outcome in terms of developing veterinary services where they are needed. We were pleased to note that the submission also stimulated contact with another faculty member in a similar situation.

As ophthalmologists and recognising the link between continuing professional development (CPD) and patient safety, Mack and colleagues (2017) undertook a review of possible frameworks for addressing the difficulty of providing CPD in low-resource settings. Their article summarises the pros and cons of five CPD models: Twinning, greenfield, formal and informal (telemedicine) programmes guided by international medical bodies and programmes directed by regulators. Mack and colleagues (2017) suggest, however, that further studies are warranted as there is little evidence of the success of any of the approaches. In the online discussion that followed, it was highlighted that all of the listed models involve external intervention and thus a key factor in their success would be one of sustainability, which necessarily requires local ownership. The article also highlights the challenges and benefits for high-resource countries when collaborating in such CPD programmes. Benefits included more ‘frugal educational innovation’ (our term), job satisfaction and the development of new skills.

Tibyampansha and colleagues (2017) from Kilimanjaro Christian Medical University College (KCMUCo, Tanzania) in partnership with Duke University (USA) describe their systematic approach to successfully implementing a learning management system (LMS) to alleviate some of the pressure associated with increasing class sizes, faculty shortages, inadequate infrastructure and limited financial resources. By strategically managing the change, which involved considerable staff and student development as well as having a ‘Plan B’, the LMS has been integrated across the entire KCMUCo community. It has been incorporated into the curriculum, facilitating the delivery of team-based and problem-based learning and also allows for online assessment.

In a submission entitled *“Pondering over medical education in the desert”*, Morikawa (2017) who was visiting Sudan as an external examiner, reflects on the situation in Family Medicine in that country, where 10 family physicians are responsible for training 6 000 future colleagues. In a politically troubled country with a severe ‘brain drain’, those 10 family physicians were debating technology as a possible solution. In his reflective submission, Morikawa (2017) concludes that irrespective of where medical students train, while technology has a place, nothing can replace the hands-on bedside experience for learning medicine. The ongoing challenge, particularly in low-resource settings, however, is providing adequate clinical supervision. Morikawa (2017) did not address this in his reflection.

Cartwright and colleagues’ (2017) submission draws attention to the many difficulties encountered by health care providers involved in re-aligning and delivering a medical curriculum in times of budgetary cuts. In their Twelve Tips approach, they suggest how careful planning and specific strategies can overcome, or at least circumvent, restricted finances and the lack of personnel expertise. In their view, strategic planning by motivated and interested faculty can produce results. The long-term sustainability of their suggestions is, however, yet to be realised.

Biswas and Tzadok’s (2017) letter entitled *“The bedside as a difficult learning environment: Overcoming the reluctance to see patients”* makes the case for the bedside providing students the opportunity to learn from and about patients and their families. To date, in our deliberations and discussions around *“Medical Education in Difficult Circumstances”*, we have focussed on issues such as the lack of resources (a widespread phenomenon) and perhaps ‘broken’ systems (e.g. a poorly functioning health care system or a toxic institutional culture). Biswas and Tzadok’s (2017) submission reminds us, however, that (like many students and medical educators) patients too may experience ‘difficult circumstances’. While any hospital stay is likely to be ‘difficult’ for most patients (e.g. unfamiliar health care setting, navigating complex management and treatment protocols) and their carers (e.g. long hours), one of the authors (Tzadok, a student) writes that learning at the bedside allows one to treat the ‘whole’ patient, which includes his or her mental well-being as well as the impact of the illness on the family.

Two papers addressed the response of medical educators to adverse weather conditions. In the first, Barzansky and Hash (2017) describe how, during Hurricane Katrina (August 2005), the Liaison Committee on Medical Education (LCME), the accrediting body of US MD programmes, has in-built support mechanisms to ensure maintenance of medical education standards (e.g. student and faculty well-being) for colleges in crisis, either because of *unanticipated* emergencies such as natural disasters or *ongoing* difficult circumstances such as difficulty in recruiting and retaining faculty. The *Take Home Messages* provide considerable direction for other fledgling accreditation bodies. Two of their *Take Home* messages are particularly important: Each medical school should have contingency disaster management plans (i.e. *What if..?* scenarios) and the value of early reporting of crises (and their management) to the accreditation body.

In the second paper, Niedl and colleagues’ (2017) case study describes the impact of adverse weather conditions on the interview-driven selection process at an American medical school, where technology helped rather than hindered the final outcome. At short notice and in the relative comfort of interviewers’ and candidates’ homes or hotel rooms, the interviews were completed with no negative effect on applicants’ acceptance. Although this paper highlighted how technology can be utilised to deal with a sudden and unexpected ‘difficult circumstance’, an additional online discussion point was the potential for more widespread use of ‘digital interviewing’ to reduce costs for applicants and colleges. There would also be environmental benefits as applicants would not need to travel for a physical face-to-face interview. How this would work for the Multiple Mini Interview (MMI) approach would, however, require considerable planning (with a back-up plan).

## Other outcomes

At the same time as the *MedEdPublish* themed edition was running, one of the authors (MM) created a Project on *ResearchGate* ^TM^, which attracted a reasonable following (16 followers, 182 reads; 22 March 2017). As the themed *MedEdPublish* articles appeared online, they were uploaded to the Project. Although this outlet did not appear to have influenced the number of *MedEdPublish* submissions, as authors, we hope that *ResearchGate* has not only served as a portal to showcase *MedEdPublish* but more importantly is an avenue to disseminate our findings more broadly, alerting a range of communities to some of the difficulties faced in medical and health care across the globe. Many of the difficulties in health professions’ education are not unique (e.g. natural disasters, austerity, low-resource settings) and through collaboration and wider communication, we may identify strategies and solutions not yet widely applied in health professions’ education.

## Conclusions

What has emerged from our interactions and discussions with colleagues, students and health care practitioners (but not patients, with whom we still need to engage) from the four corners of the world is that ‘difficult circumstances’ are ubiquitous. A ‘difficult circumstance’ ranges from one person’s perception of a toxic institutional culture of bullying and sexism to a medical school trying to graduate doctors in war-torn Syria ( McLean, McKimm & Gibbs, 2016). ‘Difficult circumstances’ may thus exist at the level of the *individual* (e.g. patient, student, educator, clinician), at the level of an *organisation* (e.g. health care facility, university or college) or at the *whole country or region level* (e.g. a failing health care system, war, conflict, epidemics, natural disasters, refugees). Solutions may be simple, such as the vulnerable individual seeking employment in a more amicable environment, while others such as the events in the Middle East are largely beyond our control as health professional educators. We must, however, continue to support colleagues, students and patients who may be innocent bystanders.

## Moving forward.….

A follow-up workshop is planned for AMEE 2017 in Helsinki in which we hope to bring together students and educators to discuss possible solutions to some of the ‘difficult circumstances’ that have been identified and, from a practical point of view, connect people and organisations to implement some of the potential solutions. We are also hoping to solicit greater input from students (e.g. through the International Federation of Medical Students’ Associations, IFMSA) as it is they who are currently learning about or may be experiencing ‘difficult circumstances’ and who may, in a few years, find themselves working in such circumstances. We also need to move from discussing issues to taking action. Data collected from various sources over the past 18-24 months (i.e. MedEdWorld survey, 2016 AMEE Conference, *MedEdPublish* themed submissions, 2017 AMEE Conference, personal input from individuals) will be collated and analysed and submitted to *Medical Teacher* in due course.

While our collaborations with the medical and health professions’ community have identified a range of current ‘difficult circumstances’ for which we seeking collaborative solutions, we need to be mindful of future challenges. We are living in difficult times - economically, politically and environmentally. As we tackle the current ‘difficult circumstances’ and ‘wicked’ problems, we should also be engaging in meaningful conversation about forthcoming anticipated ‘difficult circumstances’ (e.g. the impact of climate change) so that we can be pro-active and mitigate issues before they become problems. Not too long ago, we were asked to train graduates for an unknown future. Now, we need to train and educate our graduates for future that is more certain ( *albeit* less stable and more complex) - the consequences of climate change, religious fundamentalism, governments’ reactions and the rise of populism and conservatism. Amongst all this turmoil, the constant ‘known’ is that there will be change. It is thus up to us individually and collectively to ensure that this change is for the better. This we can do through collaboration and through sharing our experiences and resources to train graduates who better understand the causes and consequences of (ill)-health, the widening gap between those who have and those who have not and to advocate on behalf of those who might not have a voice.

## Take Home Messages

‘Difficult circumstances in medical and health professions’ education are ubiquitous.

They may exist at the level of the individual, an organisation or at a country or regional level.

We need to work collaboratively to find and implement solutions to some of our ‘wicked’ problems.

## Notes On Contributors

Prof Michelle McLean, MSc, PhD, Med, is the Academic Lead for Problem-based Learning (PBL) in the undergraduate medical programme at Bond University, Gold Coast, Australia. Having worked on three continents in three very different contexts, Michelle’s interests include diversity in learning and teaching and preparing the future medical workforce for an increasingly complex world. Her interest in *“Medical Education in Difficult Circumstances”* stems from growing up in South Africa during Apartheid and being in an academic position when transformation of higher education was required to address decades of inequity.

Professor Trevor Gibbs, MD, SFHEA, DA. FAcadMED, MMedSc, FRCGP, FAMEE, is an independent Professor and Consultant in Medical Education and Primary Care and Honorary Professor at Sun Yat-Sen University, Guangzhou, China. As Deputy Editor of *Medical Teacher*, he is responsible for the development of the AMEE Guides, the BEME Guides and the Medical Education around the World Series. His experience in General Practice and interest in medical education have provided him the opportunity to develop curricula in many parts of the world, specifically in those regions in which medical and health care education is often a challenge. He has a special interest in the social accountability of medical schools.

Professor Judy McKimm, MBA, MA(Ed), BA(Hons), PGDip (HSW), SFHEA, FAcadMed, is the Director of Strategic Educational Development and is also Professor of Medical Education in the College of Medicine, Swansea University. Judy initially trained as a nurse and has an academic background in social and health sciences, education and management. She is programme director for the Leadership Masters at Swansea and Director of ASME’s International Educational Leadership programme.

## Declarations

As editor and co-editors of the January-March 2017 theme ‘Medical Education in Difficult Circumstances’ we acknowledge a conflict of interest in writing this closing commentary.
