# Supplementary material for: Concluding commentary: Medical Education in Difficult Circumstances
Source: MedEdPublish (2016). 2017 Mar 24;6:62. [Version 1] doi: 10.15694/mep.2017.000062 (PMC10885249; doi:10.15694/mep.2017.000062)
Supplement: Peer review history

# Reviewer report: Reviewer response for version 1

**DOI:** 10.21956/mep.19468.r28712

**Published:** 2017-04-02

**Authors:** Ronald M Harden

**Affiliations:** 1 AMEE

This review has been migrated. The reviewer awarded 5 stars out of 5 Delivering a medical education programme is not easy whether at an undergraduate, postgraduate or CPD level. We are often focussed on local difficulties or problems. This series of articles beautifully summed up in a closing editorial does us a service by encouraging us to take a wider perspective and look at our own position in a broader context. . We all have something to learn from these case studies.

Reviewer Expertise:

NA

No decision status is available

# Reviewer report: Reviewer response for version 1

**DOI:** 10.21956/mep.19468.r28714

**Published:** 2017-03-30

**Authors:** Yingzi Huang

**Affiliations:** 1 The First Affiliated Hospital, Sun Yat-sen University

This review has been migrated. The reviewer awarded 5 stars out of 5 It’s a thought provoking commentary and it seems to be a topic I can’t circumvent as a Chinese. Back from the first AMEE conference in China, I was amazed by the big success of it and at the same time can’t help pondering over this question, -what next for the difficult circumstances of medical education in China? Due to its rapidly aging population, the healthcare system in China is bound to face tremendous change, which inevitably involves medical education as a crucial component. Lacking effective primary care, the simultaneously under-served and over-served population now deserves a holistic structural approach to fundamental reform rather than an opportunistic one, which has been common in China previously.Professor Trevor Gibbs and his team have been committed to helping China for many years, wearing the scars caused by countless local difficulties. As the AMEE Ambassador, I witnessed almost all the ups and downs and it makes me more than happy to see fruitful outcomes emerging now in Guangzhou, where the establishment of AMEE office in China is taking place. The worthwhile take-home messages from the past exciting encounter might be: structural approach to address local needs, local leadership and ownership towards ‘co-construction’, early reporting of possible challenges, step-wise reform avoiding radical ‘revolution’, etc. The commentary is thus a helpful summary and guidance for us to share experience and gain insights for our next steps for tomorrow’s doctors in China. Further in-depth discussions of this topic are certainly welcome in Helsinki conference soon, - together we learn from all parts of the world.

Reviewer Expertise:

NA

No decision status is available

# Reviewer report: Reviewer response for version 1

**DOI:** 10.21956/mep.19468.r28713

**Published:** 2017-03-26

**Authors:** Richard Hays

**Affiliations:** 1 James Cook University

This review has been migrated. The reviewer awarded 5 stars out of 5 I would like to thank the theme editors for bringing to publication several papers that describe the breadth, and sometimes depth, of the discussion about medical education in difficult circumstances. I suspect that we have only scratched the surface and I would like to see the discussion continue through further papers. While the original thinking was about medical education in low resources setting, it has become clear that there are other difficult circumstances - natural disasters, political and military conflicts, under-served populations and extreme health workforce shortages are examples - that add complexity and impede the supply of resources, even if only for a period of time, This concluding commentary summarises the key issues very well and provides a platform for future discussions.

Reviewer Expertise:

NA

No decision status is available
